# A self-powered and self-sensing knee negative energy harvester

**DOI:** 10.1016/j.isci.2024.109105

**Published:** 2024-02-03

**Authors:** Daning Hao, Yingjie Li, Jiaoyi Wu, Lei Zeng, Zutao Zhang, Hongyu Chen, Weizhen Liu

**Affiliations:** 1 School of Mechanical Engineering, Southwest Jiaotong University, Chengdu 610031, China; 2 Chengdu Technological University, Chengdu 611730, China; 3 Tangshan Institute of Southwest Jiaotong University, Tangshan 063008, China; 4 School of Information Science and Technical, Southwest Jiaotong University, Chengdu 610031, China; 5 School of Design, Southwest Jiaotong University, Chengdu 610031, China

**Keywords:** Physics, Engineering, Energy engineering

## Abstract

Wearable devices realize health monitoring, information transmission, etc. In this study, the human-friendliness, adaptability, reliability, and economy (HARE) principle for designing human energy harvesters is first proposed and then a biomechanical energy harvester (BMEH) is proposed to recover the knee negative energy to generate electricity. The proposed BMEH is mounted on the waist of the human body and connected to the ankles by ropes for driving. Double-rotor mechanism and half-wave rectification mechanism design effectively improves energy conversion efficiency with higher power output density for more stable power output. The experimental results demonstrate that the double-rotor mechanism increases the output power of the BMEH by 70% compared to the single magnet-rotor mechanism. And the output power density of BMEH reaches 0.07 W/kg at a speed of 7 km/h. Furthermore, the BMEH demonstrates the excitation mode detection accuracy of 99.8% based on the Gate Recurrent Unit deep learning model with optimal parameters.

## Highlights

• The HARE principle for designing human energy harvesters is proposed
• The biomechanical energy harvester (BMEH) only recovers knee negative energy
• Double-rotor and half-wave rectification mechanism enhance the power of BMEH
• Excitation mode monitoring based on optimal parameter deep learning model

## Graphical abstract

**Figure:**
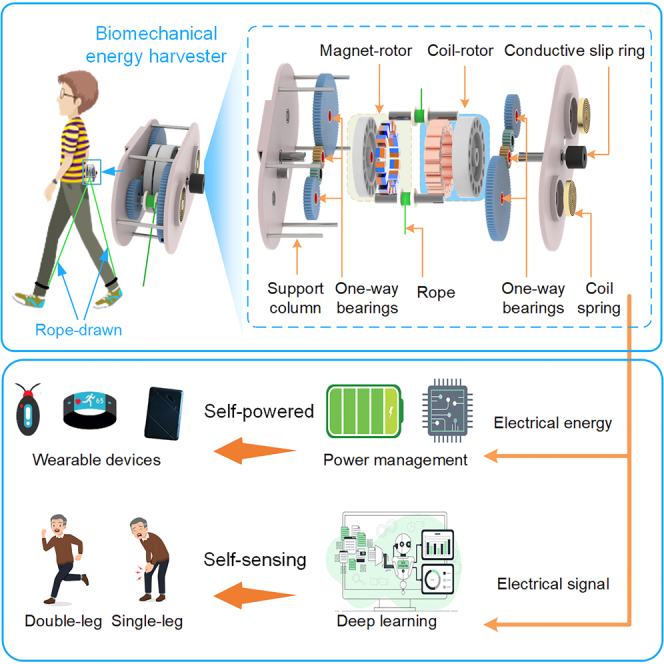

## Introduction

Wearable devices are electronic devices that are equipped on the body, and the first wearable device was reported in 1961. In today’s era of artificial intelligence (AI) and the IoT, wearable devices are seeing rapid growth.^1^^,^^2^^,^^3^ Wearable devices rely on batteries for power.^4^ However, for workers who are on the go for long periods, batteries with limited capacity need to be replaced or recharged frequently, which hinders the convenience of wearable devices.^5^ Self-powered technology based on energy harvesting is a promising solution to the endurance problem of wearable devices.^6^^,^^7^^,^^8^^,^^9^

**Figure 1:**
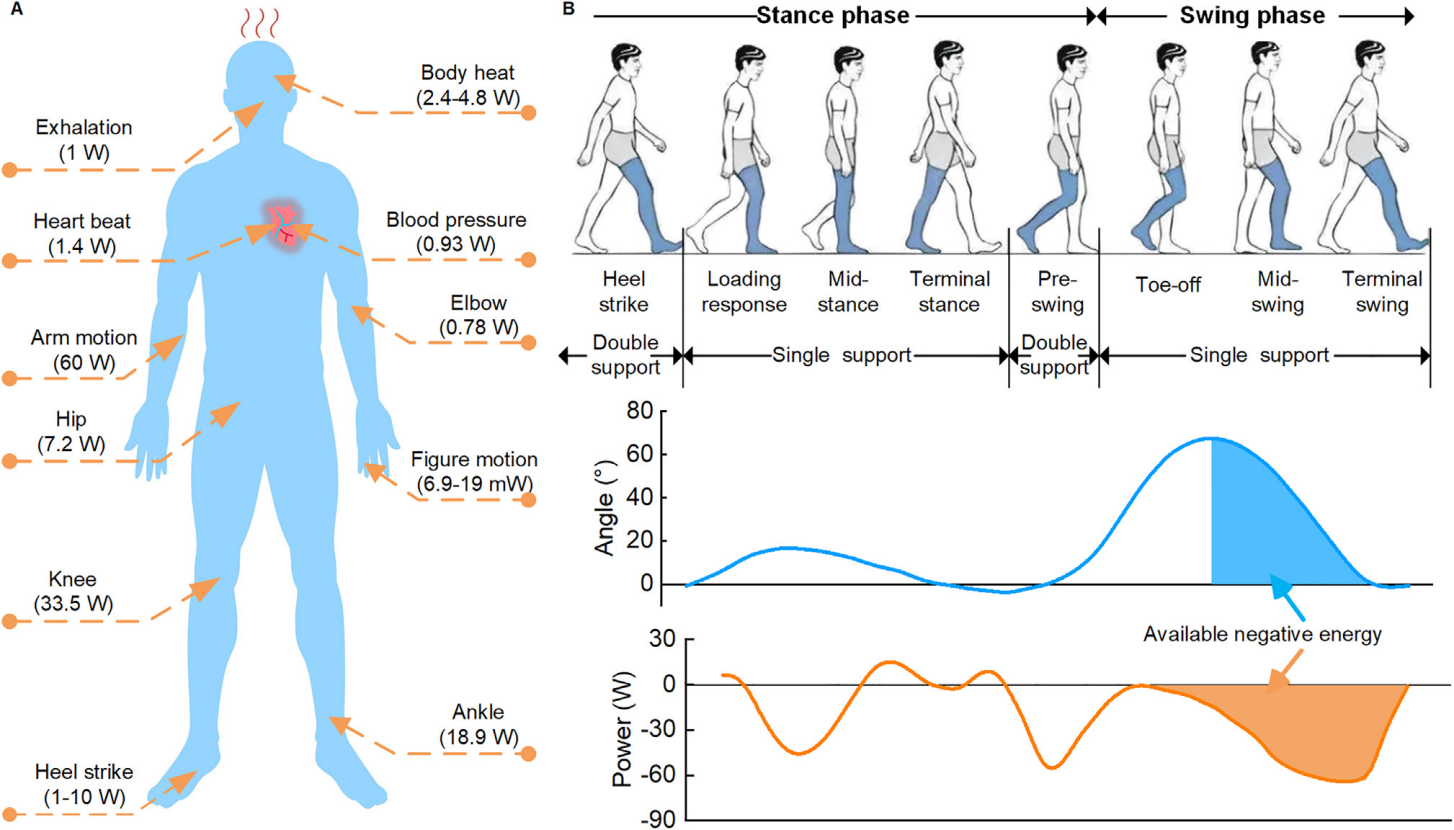
Analysis of human energy and knee joint (A) Distribution of available human energy.^11^^,^^12^^,^^13^ (B) Characteristics of the human knee joint during the normal walking cycle.^14^^,^^15^

The human body contains a large amount of recoverable energy and generates electricity to power wearable devices through self-powered technology.^10^ The available energy of the human body is shown in Figure 1A, and includes the kinetic energy of the limbs, the kinetic energy of the joints, thermal energy, etc. The energy available at the knee joint reaches 33.5W,^11^^,^^16^ which is abundant compared to other parts of the human body. Most of the wearable devices are milliwatt or microwatt level, so there is potential to recover energy from the knee joint to generate power for wearable devices. Changes in knee motion, including joint angles and energy, are cyclical. Figure 1B illustrates the characteristics of knee motion during a gait cycle. It can be found that the swing phase has the greatest knee joint angle change.

Muscles can do both positive and negative work in the process of movement. Positive work means that the muscle does centripetal movement, while negative work means the muscle does centrifugal movement. The reported studies of knee energy harvesting include recovering full process energy and recovering negative energy of the knee joint. Chen et al.,^17^ used a gear mechanism with single bearings to convert knee flexion and extension into unidirectional rotational motion of an electromagnetic generator with an average power of 3.6W. To reduce the weight of the energy harvester, Fan et al.,^18^ used a cable-pulley mechanism instead of a gear set for mechanical rectification. The proposed energy harvester achieved a maximum power of 4.1 W. Gao et al.^11^ proposed a lower limb system through the integration of a piezoelectric array generator and a triboelectric nanogenerator (TENG). The slider mechanism transforms the three-dimensional spatial motion of the knee joint into a one-dimensional linear slide and generates electrical energy using an array of piezoelectric sheets. In addition, the R-TENG is used for self-sensing. Full-process energy harvesting can fully justify the full process of gait mechanical energy generation with high output power. The performance of energy harvester affected by the carrying cost of energy harvester and the cost tied to changes in walking mechanics.^19^ However, harvesting positive energy may increase muscle energy consumption, and improve walking resistance and human metabolism. This is also contrary to the evolutionary direction of lowering metabolism in humans.^20^ Therefore, the harvesting of positive energy at the knee joint for power generation is not advocated.

In contrast to the recovery of positive energy, the recovery of negative energy is beneficial in reducing the total metabolic cost.^21^ Xie et al.,^22^ proposed a spring-damped machine based on a spiral spring and a generator, which effectively reduces the moment and power of the knee joint when doing negative work. This energy harvester can reduce the metabolic cost by 3.6% at a walking speed of 4.2 km/h and generate 5.8 W at a speed of 5.4 km/h. Comfort and safety are factors that must be considered when studying knee energy harvesters. Ren et al. studied the integration of a negative energy harvester of the knee joint into an exoskeleton. The energy harvester is mounted on the thigh and driven by ropes through the swing of the calf to achieve the conversion of negative energy to electricity.^23^ Driving the energy harvester by traction with ropes are safer and more comfortable than mounting the energy harvester directly on the knee joint. This method has also been used in some reported studies.^24^^,^^25^^,^^26^^,^^27^

The increased body load will increase the body’s metabolism.^28^^,^^29^ The increased metabolism caused by the weight of the energy harvester is an important factor to consider.^30^ In addition to the lightweight design of the energy harvester, the installation location warrants analysis. During walking, the spatial position of different parts of the body changes differently. Compared to the knee, leg, and other parts, the hip joint displacement is small.^31^^,^^32^ If the energy harvester is mounted in the hip joint can minimize the metabolism of the increased weight of the energy harvester.^33^

The existing energy harvesting methods mainly include electromagnetic,^34^^,^^35^ piezoelectric,^36^ TENG,^37^^,^^38^ etc. Among them, electromagnetic has the characteristics of simple structure, high output power, and stable power generation, and is widely used in recovering human body energy.^39^^,^^40^^,^^41^^,^^42^ Therefore, we use electromagnetic as a power generation method in this study.

Based on the previous analysis, we propose a biomechanical energy harvester (BMEH) by recovering the negative energy at the knee joint to generate electricity. In addition, there have been studies using piezoelectric generators and TENGs to use the energy harvester itself as a sensor.^43^ In this study, we attempt to utilize the BMEH to generate power for wearable devices while acting as a sensor to recognize human movement. Therefore, the proposed BMEH achieves the self-sustainable of powering wearable devices and sensing. Compared with previous studies, the innovations and contributions of this research include: (i) the proposed BMEH is mounted at the waist and driven by ropes through the swing of the calf. This mounting is more stable and comfortable than mounting the energy harvester directly at the knee joint, and also reduces the increased metabolism caused by the weight of the energy harvester; (ii) the acceleration gear set and half-wave rectification mechanism converts the ultra-low frequency motion of the human knee joint into high-speed relative rotational motion of the coils and magnets, which increases the power generation time and power generation capacity of BMEH; (iii) compared with the single-rotor, the double-rotor design can effectively improve energy conversion efficiency, with higher power output density and more stable power output; (iv) combined with deep learning to recognize the different excitation modes of BMEH to achieve self-sensing.

### Design philosophy of human energy harvester

**Figure 2:**
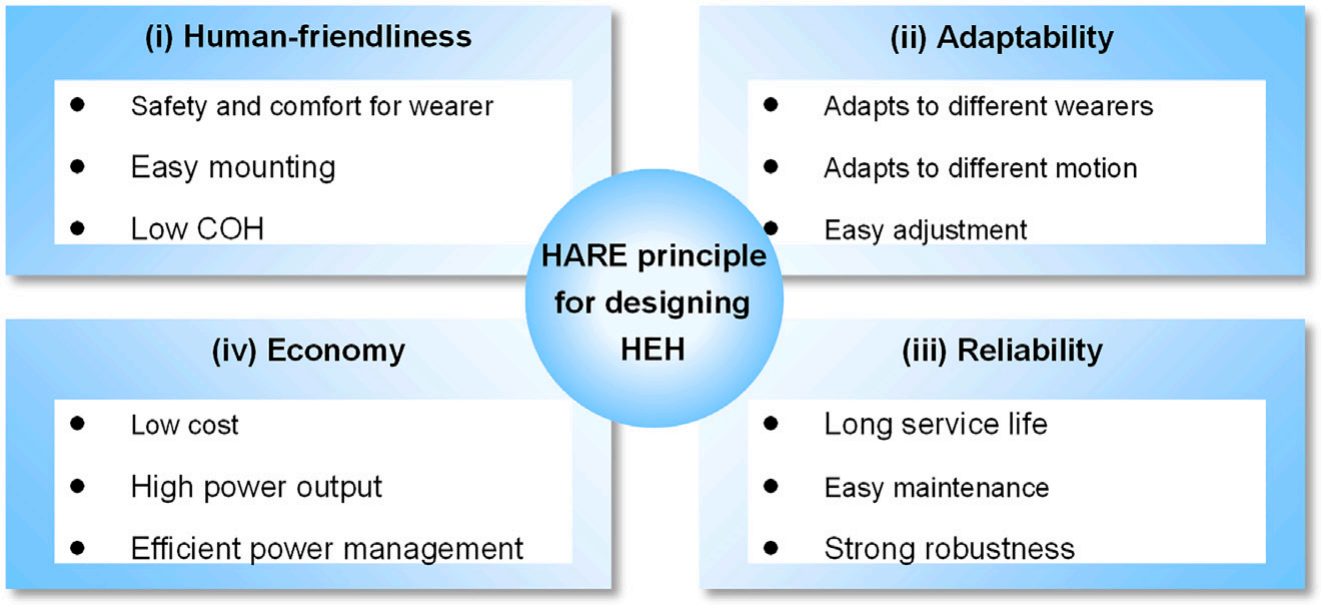
The HARE principle for designing a HEH

The human body contains a large amount of energy that can be utilized, such as thermal and kinetic energy. Recovering human body energy to generate electricity for wearable devices is promising. However, since the human energy harvester (HEH) needs to be mounted on the human body, there are some considerations when designing an HEH. Here, the HARE design principles are proposed to guide the design of a HEH. The HARE principle specifically refers to human-friendliness, adaptability, reliability, and economy. Figure 2 reveals the specific meaning of the HARE principle.

Human-friendliness is the first topic to be considered in HEH design. First, the HEH should be safe and comfortable for the wearer and not interfere with the wearer’s normal activities. Secondly, the HEH should be easy to mount, and the wearer can do the mounting and dismounting of the HEH by himself/herself. Finally, low cost of harvesting (COH) is considered and pursued in the design of HEHs.^15^^,^^44^^,^^45^

Adaptability is the second focus of research in HEH design. Human movement is irregular, in daily life, the human body carries out walking, jogging, squatting, etc. Therefore, the designed HEH should be able to work under different movements, speeds, postures, etc. of the wearer. At the same time, the HEH should also be universal, the HEH can be directly used by wearers of different heights, weights, and other characteristics without adjustment or micro-adjustment.

Reliability and economy are also aspects that must be considered in HEH design. HEH can be seen as a special wearable device. Reliability and economy are what all products must consider. On the one hand, HEH is required to have good power generation capacity to meet the demand of wearable devices. At the same time, HEH should have good robustness to ensure that it can maintain normal operation under long-time use and various motion conditions and external environments. Excellent reliability and economic performance contribute to the HEH being put into real use and popularized.

### System design and working principle

In this section, the proposed BMEH based on the HARE principle is presented. The structure and working principle of BMEH are described in detail.

#### Overview

**Figure 3:**
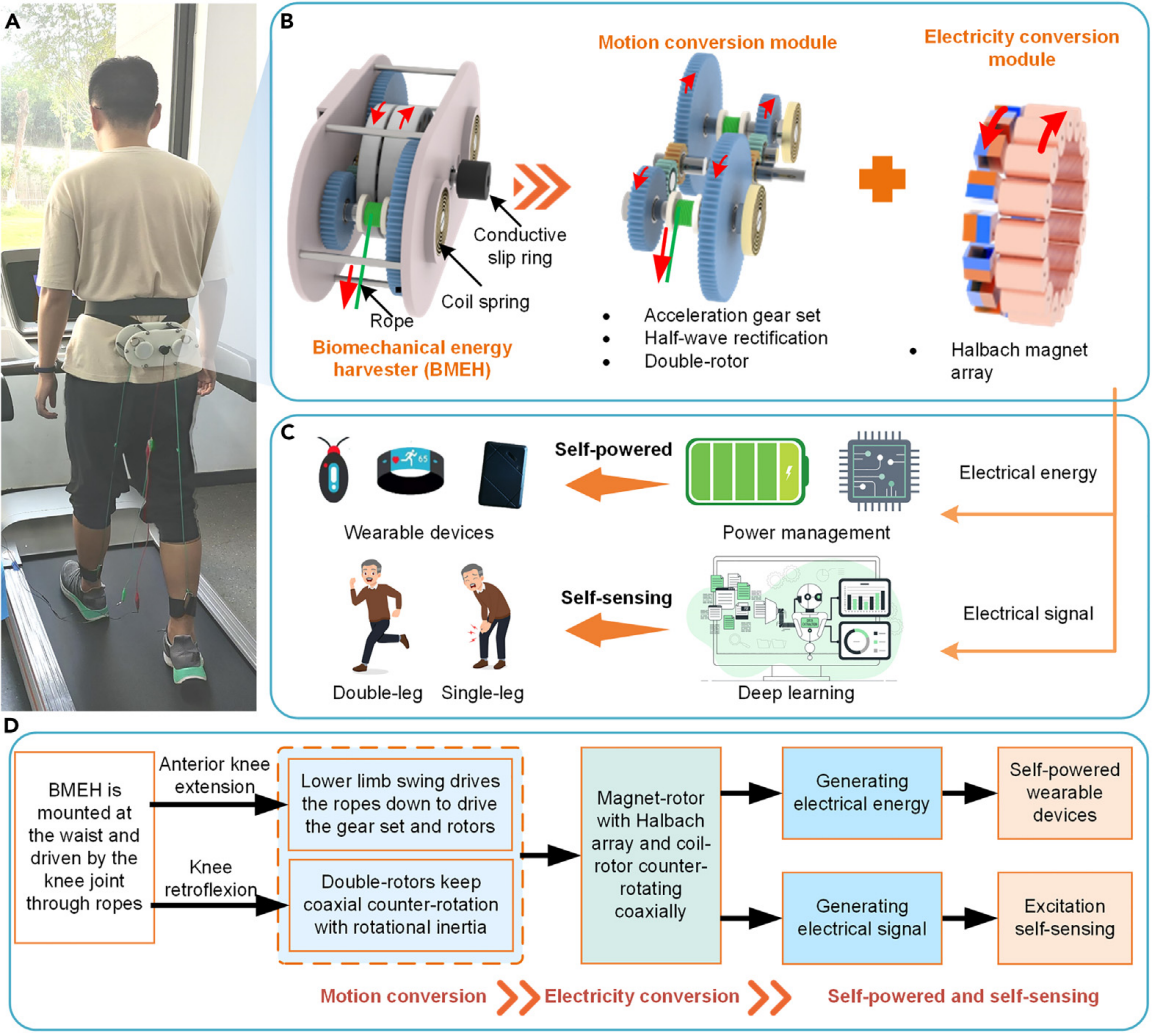
Overview of the proposed BMEH (A) Model wears the BMEH. (B) Components and characteristics of BMEH. (C) Schematic of self-powered and self-sensing. (D) Work flow.

The proposed BMEH includes a motion conversion module and an electricity conversion module, which in combination with an energy storage module can realize the recovery of negative energy from the knee joint to power the wearable devices, as shown in Figure 3. The motion conversion module converts the ultra-low frequency knee extension motion into the high-frequency rotation motion of the double-rotor. The electricity conversion module based on Halbach magnet arrays generates electricity. The BMEH generates unstable alternating current (AC) power, which cannot directly power the wearable devices. Voltage stabilization and rectification is the necessary process for BMEH to generate electricity for wearable devices. Firstly, a rectifier bridge converts the AC power generated by BMEH to direct current (DC) power. Then the output voltage is adjusted to the voltage required by the wearable device using a voltage regulator circuit. Finally, the generated electricity is stored in a capacitor to power the wearable devices.

The BMEH is mounted on the waist of the human body and connected to the calves through ropes, and the extension motion of the knee joint is transformed into a rotational motion. When the knee joints are extended, the muscles do negative work and the BMEH recovers the negative energy generated to generate electricity. The BMEH recovers negative energy to help reduce the negative work done by the muscles and reduce muscle exertion. When the knee joints are flexed, the muscles do positive work. The ropes are retracted by the restoring force of coil springs, and the BMEH does not create resistance to knee retraction and increase muscle exertion. The rotation motion is accelerated by the acceleration gear set, and finally the half-wave rectification mechanism is used to realize the high-speed coaxial counter-rotation of the double-rotor.

Compared with the knee joint, the waist changed little in spatial position during human walking, so the metabolic increase caused by BMEH weight was small; in addition, mounting the BMEH to the waist is easier to handle and more stable. Compared with the BMEH directly mounted at the knee joint, it is more friendly to the knee joint, does not restrict the spatial movement of the knee joint, and does not cause injury or discomfort to the human body. The rope length is adjustable, making the BMEH suitable for any wearer.

#### Structure and working principle of BMEH

**Figure 4:**
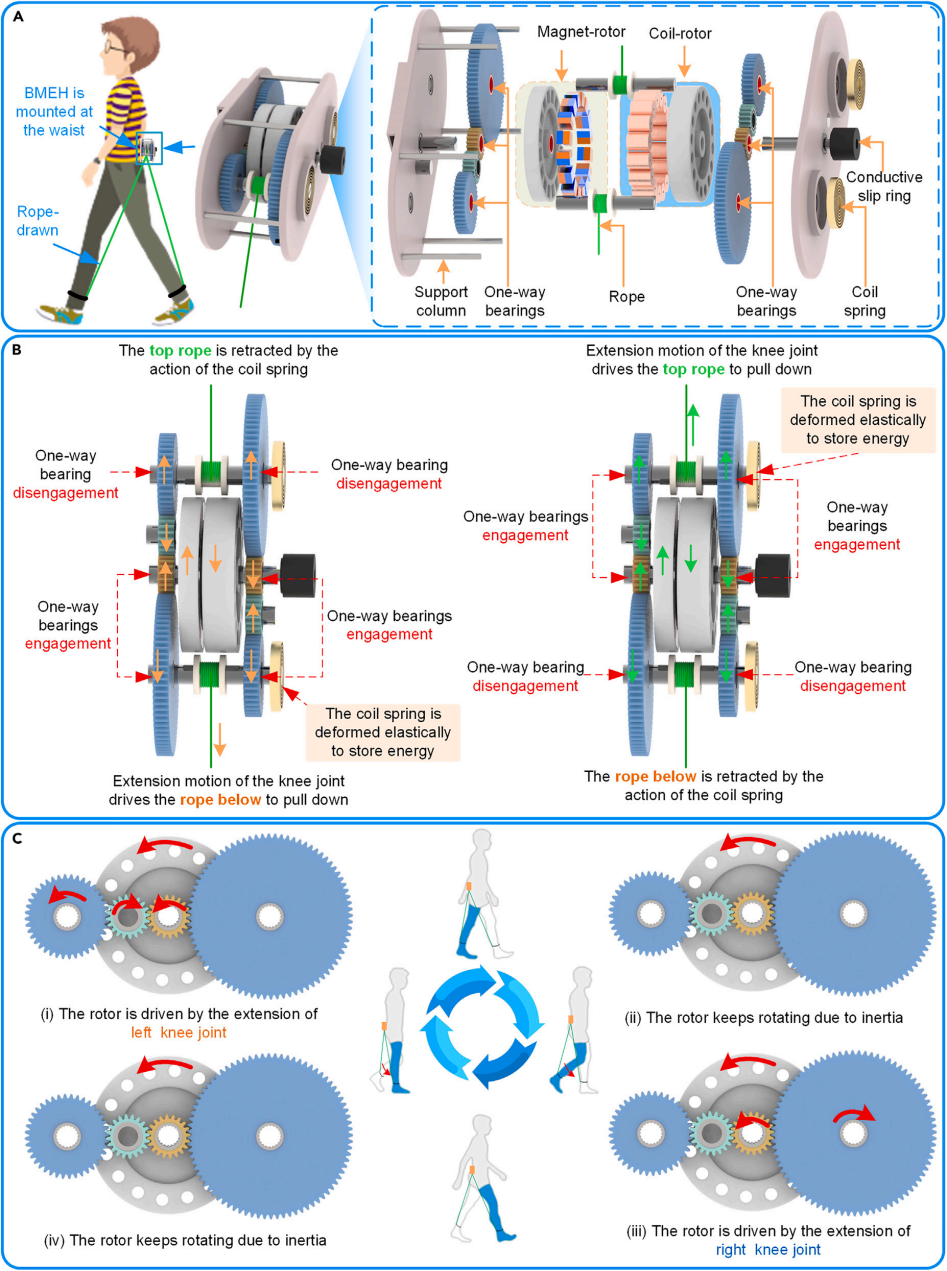
Structure and working principle of BMEH (A) Structure of the proposed BMEH. (B) Working principle of the proposed BMEH. (C) Schematic diagram of rotor keeping unidirectional rotation.

The structure and working principle of the proposed are illustrated in Figure 4. The total weight of the BMEH is 1.043 kg and the parameters of the main parameters are listed in Table 1. The structure of BMEH is shown in Figure 4A, which mainly consists of the rope, gear set, conductive slip ring, one-way bearing, magnet-rotor, coil-rotor, coil spring, support column, etc. The ropes are used to connect the calf to the BMEH, enabling the use of negative energy from knee extension to drive the gear set. The gear set converts the knee’s swing into a rotational motion of the gears and accelerates the gears utilizing a different ratio design. Some of the gears are fitted with one-way bearings inside to keep the rotor rotating unidirectionally. The magnet rotor and coil rotor rotate in coaxial counter-rotation to generate electrical energy. A conductive slip ring fixedly attached to the coil rotor ensures that the coil rotor is rotatable. The coil spring undergoes elastic deformation to store energy during knee extension and is set to release energy when the knee is flexed, utilizing the restoring force to return the ropes to their original position in preparation for the next work cycle of the BMEH. Support columns ensure the overall stability of the BMEH.

**Table 1:** Main parameters of the BMEH

Parameter	Value
Material (rotor)	Resin
Size (rotor)	*Φ* 80 mm × 13 mm
Material (magnet)	NdFeB (N35)
Size (magnet)	9 mm × 9 mm × 9 mm
Outer diameter (coil)	12 mm
Inner diameter (coil)	0.16 mm
Thickness (coil)	15 mm
Thread diameter (coil)	0.2 mm
Impedance of each coil	18 Ω
Number of teeth of the large gear	80
Number of teeth of the medium gear	40
Number of teeth of the pinion gear	20
Module of gears	1
Thickness of gears	8 mm

Figure 4B shows the working principle of the proposed BMEH. The extension motion of the knee joint drives the rope down to drive the gear rotation. The transfer of force is controlled by the engagement and disengagement of one-way bearings. When the rope is pulled down on one side, the one-way bearings inside the gears on the same side engage to transmit the force. And the coil spring on the same side deforms elastically to store energy. Meanwhile, the one-way bearings inside the gear on the other side are disengaged and do not transmit force. When the knee is in flexion, the coil spring releases energy and uses the restoring force to return the rope to its initial position in preparation for the next work cycle of the BMEH.

The unidirectional rotation of the rotor is shown in Figure 4C. In a gait cycle, the BMEH is first driven by the extension motion of the left knee joint, which generates electricity using negative knee work; when the left knee joint is in flexion, the rotor of the BMEH keeps rotating due to half-wave rectification and rotational inertia. Then the BMEH is driven by the extension motion of the right knee joint; when the right knee joint is in flexion, the rotor of the BMEH keeps rotating due to half-wave rectification and moment of inertia.

### Modeling and analysis

#### Dynamic analysis of the BMEH

The mechanical motion rectification (MMR) module converts irregular external excitation into high-speed unidirectional rotational movement of the magnet-rotor and the coil-rotor. The MMR of the magnet-rotor is the same as the MMR of the coil-rotor, both of which are composed of a primary gear system and a secondary gear system, as shown in Figure 4. According to whether the one-way bearings inside the gears I and II transmit torque, the motion state of BMEH can be divided into two types. When the one-way bearing transmits torque, the magnet-rotor and the coil-rotor rotate freely. When the one-way bearing transmits torque, the dynamics of the primary gear system and secondary gear system are as follows:

(Equation 1) $$T_{M}-T_{M1}=J_{2}{\ddot{\theta }}_{2}+J_{3}{\ddot{\theta }}_{3}+J_{4}{\ddot{\theta }}_{4}+T_{f1}$$

(Equation 2) $$T_{C}-T_{C1}=J_{1}{\ddot{\theta }}_{1}+J_{5}{\ddot{\theta }}_{5}+T_{f2}$$

*T*_*M*_ and *T*_*C*_ are the input torque provided by the knee joint to the primary gear system and secondary gear system through the rope, respectively. *T*_*M1*_ and *T*_*C1*_ are the input torque provided by the primary gear system and secondary gear system to the power generation module, respectively. *J*_*1*_, *J*_*2*_, *J*_*3*_, *J*_*4*_, and *J*_*5*_ represent the moment of inertia of gear I, gear II, gear III, gear IV, and gear Ⅴ, respectively. *θ*_*1*_, *θ*_*2*_, *θ*_*3*_, *θ*_*4*_, and *θ*_*5*_ represent the rotation angles of gear I, gear II, gear III, gear IV, and gear Ⅴ, respectively. *T*_*f1*_ and *T*_*f2*_ are the friction moments generated when the primary gear system and secondary gear system engage, respectively.

The relationship between *θ*_*1*_, *θ*_*2*_, *θ*_*3*_, *θ*_*4*_, and *θ*_*5*_ is as follows:

(Equation 3) $$\theta_{1}=\theta_{2}$$

(Equation 4) $$\theta_{3}=n_{23}\times \theta_{2}=\frac{z_{3}}{z_{2}}\times \theta_{2}$$

(Equation 5) $$\theta_{4}=n_{24}\times \theta_{2}=\frac{z_{3}}{z_{2}}\times \frac{z_{4}}{z_{3}}\times \theta_{2}=\frac{z_{4}}{z_{2}}\times \theta_{2}$$

(Equation 6) $$\theta_{5}=n_{15}\times \theta_{1}=\frac{z_{5}}{z_{1}}\times \theta_{1}$$

*z*_*1*_, *z*_*2*_, *z*_*3*_, *z*_*4*_, and *z*_*5*_ are the number of teeth of gear I, gear II, gear III, gear IV and gear Ⅴ, respectively, and the relationship is as follows:

(Equation 7) $$z_{1}=2z_{2}=4z_{3}=4z_{4}=4z_{5}$$

The friction torque *T*_*f1*_ and *T*_*f2*_ can be described as follows:

(Equation 8) $$T_{f1}=sign\left(\dot{\theta }\right)\left[T_{s1}+\left(T_{s1}-T_{C_{1}}\right)e^{-C_{1}|{\dot{\theta }}_{1}|})\right]+C_{v1}\dot{\theta }$$

(Equation 9) $$T_{f2}=sign\left(\dot{\theta }\right)\left[T_{s2}+\left(T_{s2}-T_{C_{2}}\right)e^{-C_{2}|{\dot{\theta }}_{2}|})\right]+C_{v2}\dot{\theta }$$

where, *T*_*s1*_ and *T*_*s2*_ are the separation friction torque of the primary gear system and secondary gear system, respectively. *T*_*c1*_ and *T*_*c2*_ are the Coulomb friction torque of the primary gear system and secondary gear system, respectively. *C*_*1*_ and *C*_*2*_ are the transition approximation factors of the MMR 1 module and MMR 2 module, respectively. *C*_*v1*_ and *C*_*v2*_ are the rotational frictional damping of the primary gear system and secondary gear system, respectively.

The maximum starting torque of the BMEH increases as the walking speed of the human body increases. The maximum starting torque of the BMEH was experimentally tested to be 1.2 Nm, whereas the root mean square (RMS) value of the torque of the knee joint during the gait cycle of a 70 kg body is 18.15 Nm.^18^ It can be calculated that the maximum starting torque of the BMEH is only 6.61% of this. Therefore, driving the BMEH hardly hinders people’s normal walking.

#### Simulation analysis of magnet array

**Figure 5:**
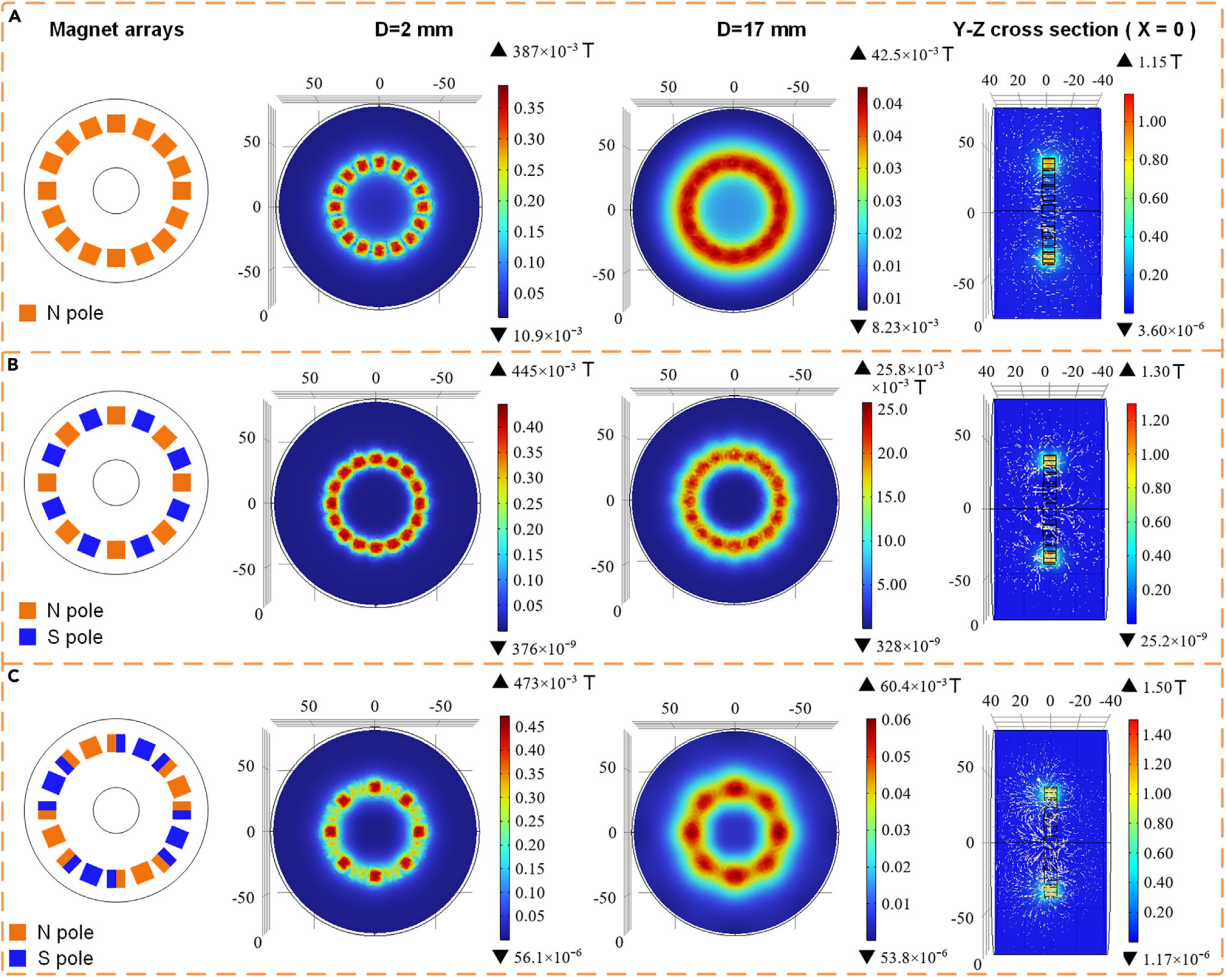
The magnetic flux density of three different magnet arrays (A) The poles of all magnets are the same. (B) The poles of two adjacent magnets are opposite. (C) Halbach array.

The magnetic flux density and distribution of magnetic induction lines are influenced by the magnet array. To obtain a better magnet array, three possible magnet arrays were analyzed using COMSOL Multiphysics. The three magnet arrays are all magnets with the same pole direction, adjacent magnets with opposite pole directions, and Halbach array. The simulation results of three different magnet arrays obtained using COMSOL Multiphysics are shown in Figure 5. The white arrows in the Figure represent the magnetic induction line. Three different magnet arrays produce different distributions of magnetic induction lines. The distance between the magnet coils of the designed BMEH is set to 2 mm.

Figure 5 demonstrates that the maximum flux density of three different magnet array. It can be found that the Halbach magnet array produces the strongest magnetic flux density for the same distance. Besides, the magnetic field distribution of the Halbach magnet array is uniform, which can reduce the interference of the magnetic field gradient on the generator and improve the stability and reliability of the generator.^46^^,^^47^ Since the magnets and coils are always parallel in this study, the Halbach magnet array is used for the proposed BMEH.

### Experimental setup

A prototype of the proposed BMEH was fabricated for experiments to verify the real performance of the designed BMEH. Several test subjects of different genders and physical characteristics were selected for testing and their consent to participate in the experiment was obtained before the experiment. Considering the variability and irregularity of human motion, we selected multiple testers for experiments. All testers were experimented on a treadmill with a speed of 1∼7 km/h. The movement state includes walking and jogging. A resistor box (SHANE ZX99-IA, measurement accuracy: 0.1%) was used to provide external load. The internal resistance of the BMEH is 288 Ω, so the external load is set to 288 Ω during the experiment for obtaining the maximum power of BMEH.^48^^,^^49^^,^^50^^,^^51^

## Results and discussion

### The electricity performance of the BMEH

**Figure 6:**
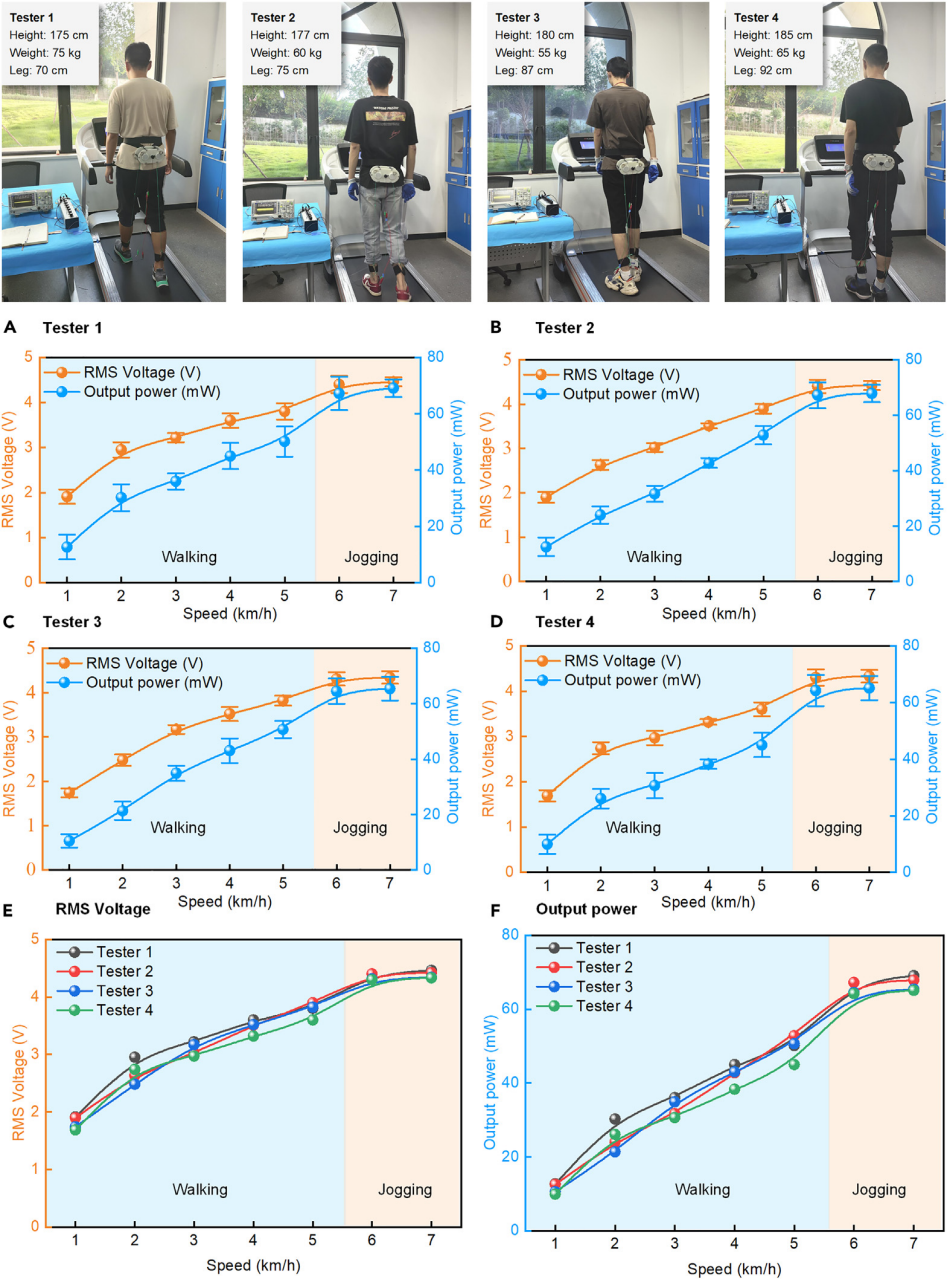
The output performance of BMEH under different testers and different motion speeds test on the treadmill (A–D) Performance of the proposed BMEH worn on four testers at different speeds. (E) RMS voltage of the proposed BMEH worn on four testers at different speeds. (F) Output power of the proposed BMEH worn on four testers at different speeds.

Four testers with different heights and weights were selected to test the electricity performance of the proposed BMEH on the treadmill. The treadmill speed was set to 1–7 km/h, where the testers walked on the treadmill at 1–5 km/h and jogged at 6–7 km/h. The test results are shown in Figure 6.

From (Figures 6A‒6D), it can be observed that the electrical performance of the BMEH has some errors under different tests. However, the error is relatively small and decreases with increasing speed. When the speed is 7 km/h, the RMS voltage error is 1.4% (for tester 3 and tester 4), and the output power error is less than 1%. There are various reasons for the errors. Firstly, human body movements are variable and irregular, making it difficult for the testers to maintain the same movement state in different tests at the same speed. Furthermore, the prototype of BMEH used in the experiment is 3D printed, and as the experiment progresses, components like gears experience wear, which affects torque transmission. Additionally, the BMEH prototype used in the experiment is manually assembled, and the assembly precision also affects the output stability of the BMEH. Finally, the accuracy of the equipment used in the experiment also affects the experimental results.

(Figures 6E and 6F) demonstrate that the RMS voltage and output power of the BMEH as a whole increase with speed and eventually converge to a stable value for all four testers. The largest range of RMS voltage variation was observed for tester 3, which increased by 2.6 V from 1.74 V to 4.34 V and eventually stabilized at 4.4 V. The output RMS voltages of tester 1, tester 2, and tester 3 tend to stabilize at 4.5V, 4.5V, and 4.4V, respectively. The output power of BMEH worn by tester 1, tester 2, tester 3, and tester 4 are 69.07 mW, 67.83 mW, 65.4 mW, and 65.1 mW, respectively. The average output power is 66.85 mW. Testers with body differences such as height and weight produce similar power output, indicating that the output power of designed BMEH is relatively stable and does not produce widely varying power output depending on the wearer. This is because only when the new excitation gives the rotational speed of the shaft connected to the rotor is greater than the rotational speed of the rotor, the one-way bearing will engage to transmit the torque, which in turn drives the rotor to rotate for power generation. In fact, during the rotation time of rotors, the rotors rotate for power generation under their rotational inertia part of the time, and the one-way bearings are in a non-engaged state, resulting in the generated torque when the knee joint does negative work not always being transmitted to the rotors.

Another obvious phenomenon is that when the tester’s motion state changes from walking to jogging, the electrical performance of BMEH is significantly improved. The output power of BMEH worn on the four testers increased by 34%, 27%, 27%, and 42%, respectively. This is because from walking to jogging, the swing frequency of the lower limbs will increase, thus increasing the excitation input frequency of the BMEH, and finally improving the power output of the BMEH.

### Performance improvement of BMEH by double-rotor

As can be seen from Figure 6, although the electricity output generated by the BMEH varies depending on the testers wearing it, the difference is not significant. Therefore, in the subsequent experiments, we conducted the performance tests of BMEH with tester 1 wearing BMEH.

**Figure 7:**
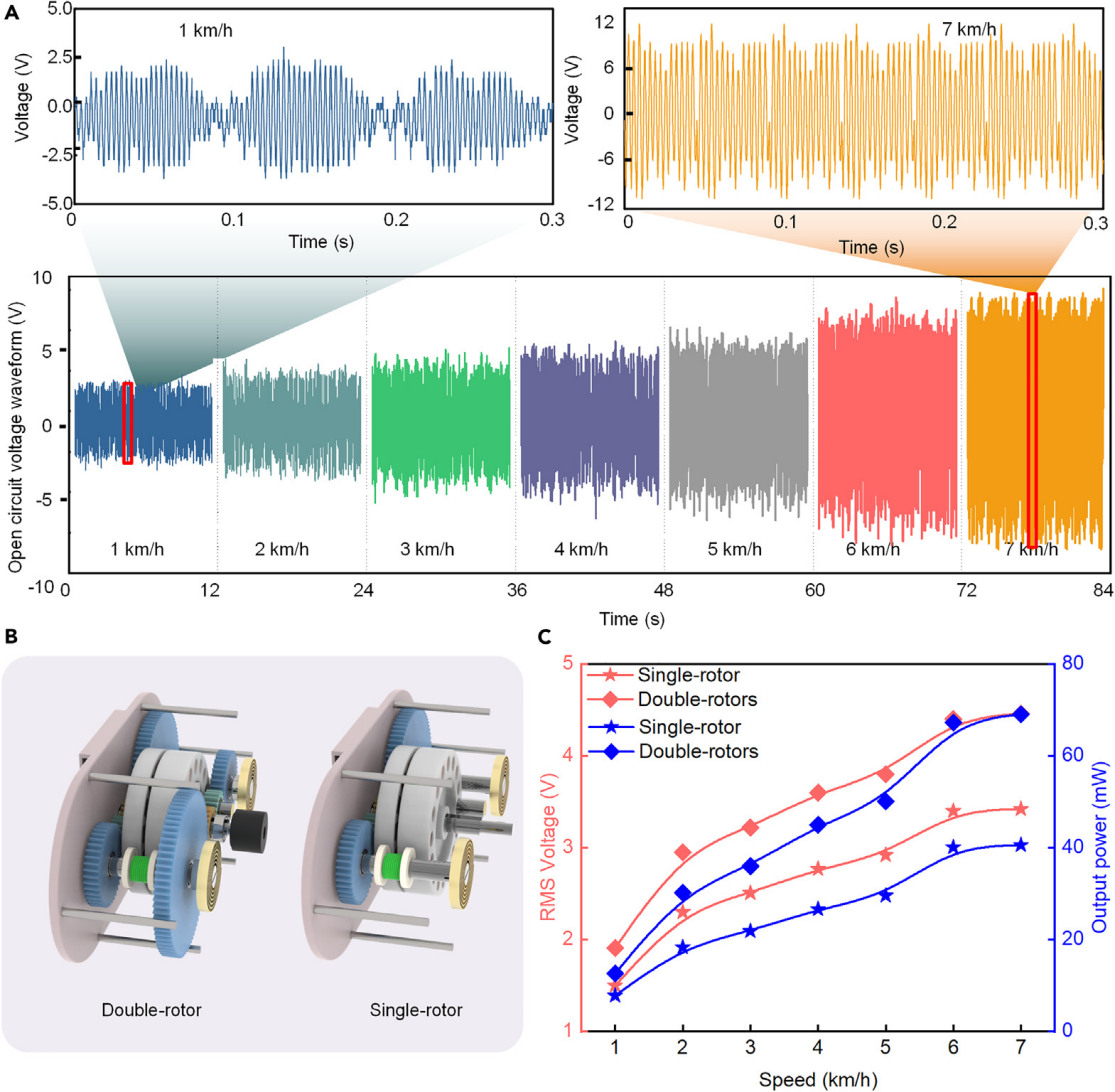
Performance improvement of BMEH by double-rotor (A) Open-circuit voltage waveform of BMEH under different speeds. (B) Single-rotor and double-rotor structure of BMEH. (C) Performance of BMEH at different speeds.

The open-circuit voltage waveforms of BMEH at different speeds are shown in Figure 7A. The peak voltage of BMEH is positively correlated with the speed. To show the voltage waveform more clearly, we selected some waveforms of BMEH at the speeds of 1 km/h and 7 km/h for analysis. It can be found that there is an obvious drop interval of the output voltage of BMEH at the speed of 1 km/h, while at the speed of 7 km/h, the BMEH always maintains a high output voltage and has no drop trend.

This is because the proposed BMEH recovers negative energy from the knee joint, which is driven by the swing of the lower limb. In general, the lower the speed of human movement, the lower the frequency of lower limb oscillation. The rotor based on the half-wave rectification mechanism has a flywheel effect and the BMEH thus maintains power generation in the absence of external excitation. At low speeds, the rotor speed decreases with time, resulting in a decrease in the voltage of the BMEH. As the speed increases, the excitation frequency increases. When the speed is 7 km/h, the rotor speed receives new excitation without obvious weakening, so the BMEH can maintain high output voltage. The voltage waveform curve in some areas is rough because the BMEH is 3D printed by resin material and has certain errors through manual assembly.

The double-rotor mechanism is an important component of the BMEH, and we investigate its electrical performance improvement compared to the single-rotor mechanism through experimental analysis. The BMEH based on the single-rotor and double-rotor mechanism is shown in Figure 7B. It can be found that the voltage and output power of both BMEHs change in the same trend, as the speed increases. When the speed of the tester’s motion state was changed from walking to jogging, the electrical output was significantly increased.

Figure 7C shows the voltage and power of BMEH with double-rotor and the BMEH with single-rotor. At a speed of 7 km/h, the voltage and output power of the BMEH with single-rotor reach 3.42 V and 40.6 mW, respectively, while the voltage and output power of the BMEH with double-rotor reach 4.46 V and 69 mW, respectively. It can be calculated that the voltage and output power of BMEH are increased by 30.4% and 70%, respectively, for the double-rotor design compared to the single-rotor. When the tester speed is 1 km/h, the double-rotor design also achieves a 27.3% and 62.1% increase in voltage and output power, respectively, compared to the single-rotor.

The BMEH with a single-rotor receives a larger torque, so the generation time is greater than that of the dual-rotor BMEH with double-rotor. However, due to the limited torque transfer capability of the one-way bearing and the fact that the double-rotor mechanism performs coaxial counter-rotation, the speed of cutting magnetic induction lines is greater than that of the single-rotor BMEH. Therefore, the peak voltage of the BMEH with double-rotor is greater than the peak voltage of the BMEH with single-rotor.

Table 2 lists some typical knee joint energy harvesters and compares them in terms of mass power density. Due to different experimental setups, it is unable to compare the power densities of different knee joint energy harvesters under the same experimental excitation. In different literatures, the power of similar excitation conditions is chosen for comparison as much as possible. It can be found that the BMEH proposed in this study has a high power density. The power density of the knee energy harvesters reported in Rome et al.,^7^ and Shepertycky et al.,^26^ are higher than that of this study. It is because they are generated by commercial generators, and although they have high power density, they are also heavy, which has an impact on human comfort.

**Table 2:** Comparison of typical knee joint energy harvesters

Reference	Year	Power density (W/kg)	Experimental excitation
Rome et al.^7^	2005	0.13	5.6 km/h
Shepertycky et al.^26^	2021	0.23	Walking
Gao et al.^11^	2021	0.02	0.75 Hz
Wang et al.^52^	2022	0.04	8 km/h
Zhang et al.^16^	2023	0.002	1 Hz
Kong et al.^51^	2023	0.05	2 Hz
This work		0.07	7 km/h

### The performance of practical applications

**Figure 8:**
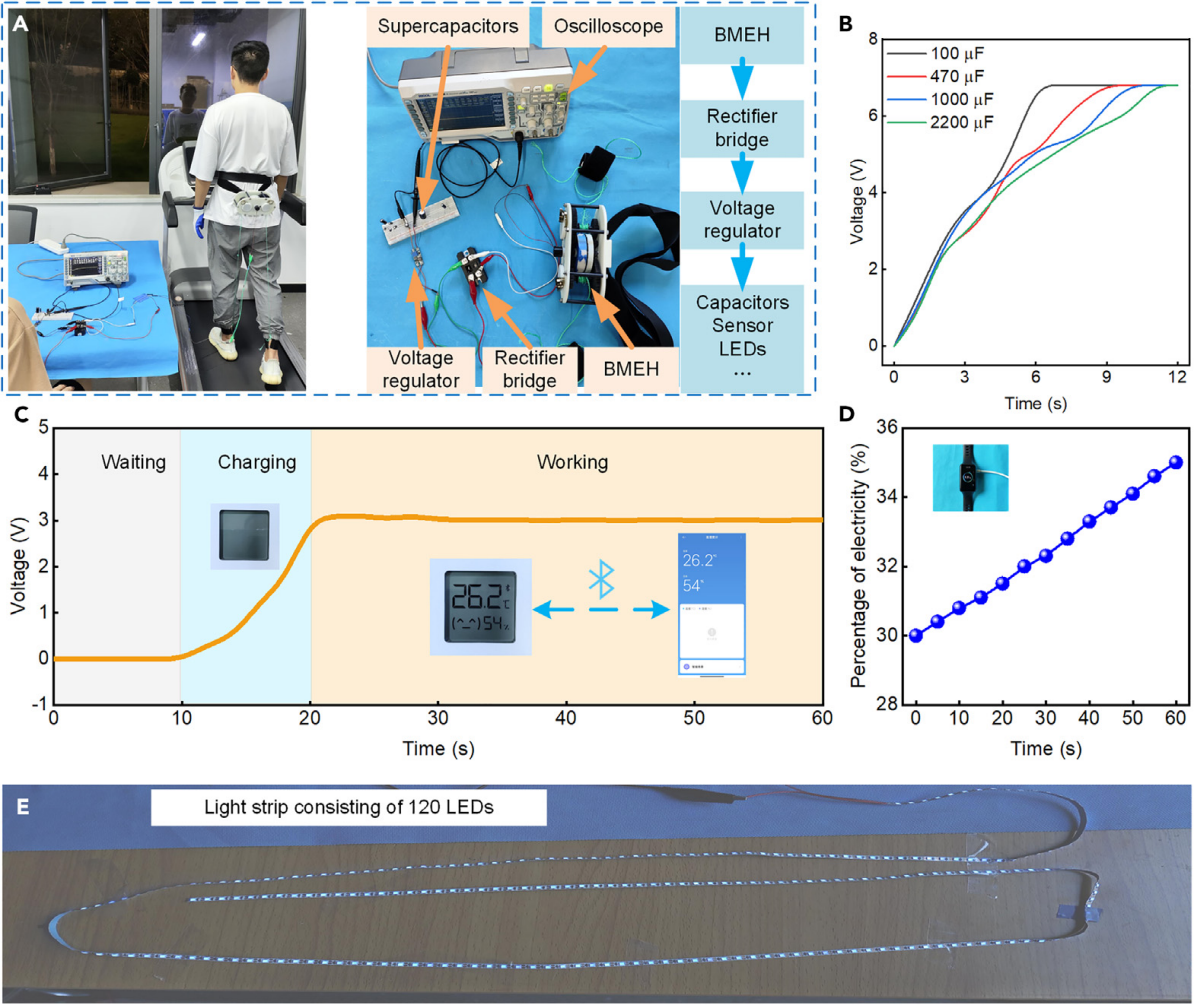
The practical application test of the BMEH (A) Overview of the practical application test. (B) Charging four different capacitors. (C) Charging a 1000 μF capacitor and powering a wireless temperature and humidity sensor. (D) Charging the bracelet. (E) Lighting strip consisting of 120 LEDs.

To verify the usability of the designed BMEH, we conducted some practical tests, as shown in Figure 8. During the experiments, the tester speed was set to 4 km/h, which is the normal walking speed of people. The tests included charging the capacitor, powering the temperature and humidity sensor, and lighting the light-emitting diodes (LEDs). Figure 8A illustrates the overview of the practical tests. The electricity generated by the BMEH is AC, and the generated AC is converted to DC by a rectifier bridge. The rectified DC voltage is then regulated through a voltage regulator, which then supplies power to the capacitors, sensors, LED lights, etc. When supplying power to the devices, it is necessary to first adjust the output voltage of the voltage regulator module to the rated voltage of the devices.

Figure 8Bshows the charging curves of BMEH for four different capacitors. The output voltage of the voltage regulator module is set to 6.8 V. It can be found that the BMEH charges 100 μF, 470 μF, 1000 μF, and 2200 μF capacitors to 6.8V in 6 s, 9 s, 10 s, and 11 s, respectively. In general, the lower the capacitance, the faster the charging speed. In some intervals, the charging rate of the capacitor with a small capacity is lower than that of the large capacitor. The reason is that the charging experiments for the four capacitors were performed separately, and the tester cannot keep the gait the same, resulting in some differences in the BMEH generation. We tested it by driving a temperature and humidity sensor, charging a bracket, and lighting up LEDs for example. As can be seen in Figure 8C, the power generation of BMEH can drive the temperature and humidity sensor after charging the capacitor at 1000 μF for 20 s. When driving the temperature and humidity sensor, the output voltage of the voltage regulator module is adjusted to 3 V. Furthermore, the electronic bracelets are common wearable devices with various functions such as location tracking and heart rate monitoring. A Huawei Band 7 bracelet was selected for charging with BMEH. The tester wearing BMEH walked at 4 km/h for 1 h during the experiment, and the charging result is shown in Figure 8D. When charging the bracelet, the output voltage of the voltage regulator module is adjusted to 5 V. The power of the bracelet’s battery was charged from 30% to 35%. The power generated by BMEH can last the bracelet up to 16.8 h in a typical usage scenario. In addition, the electricity generated by the BMEH can light up a strip of 120 LEDs, as shown in Figure 8E. And the rated voltage of the strip is 5 V.

### Fatigue experiments of BMEH

**Figure 9:**
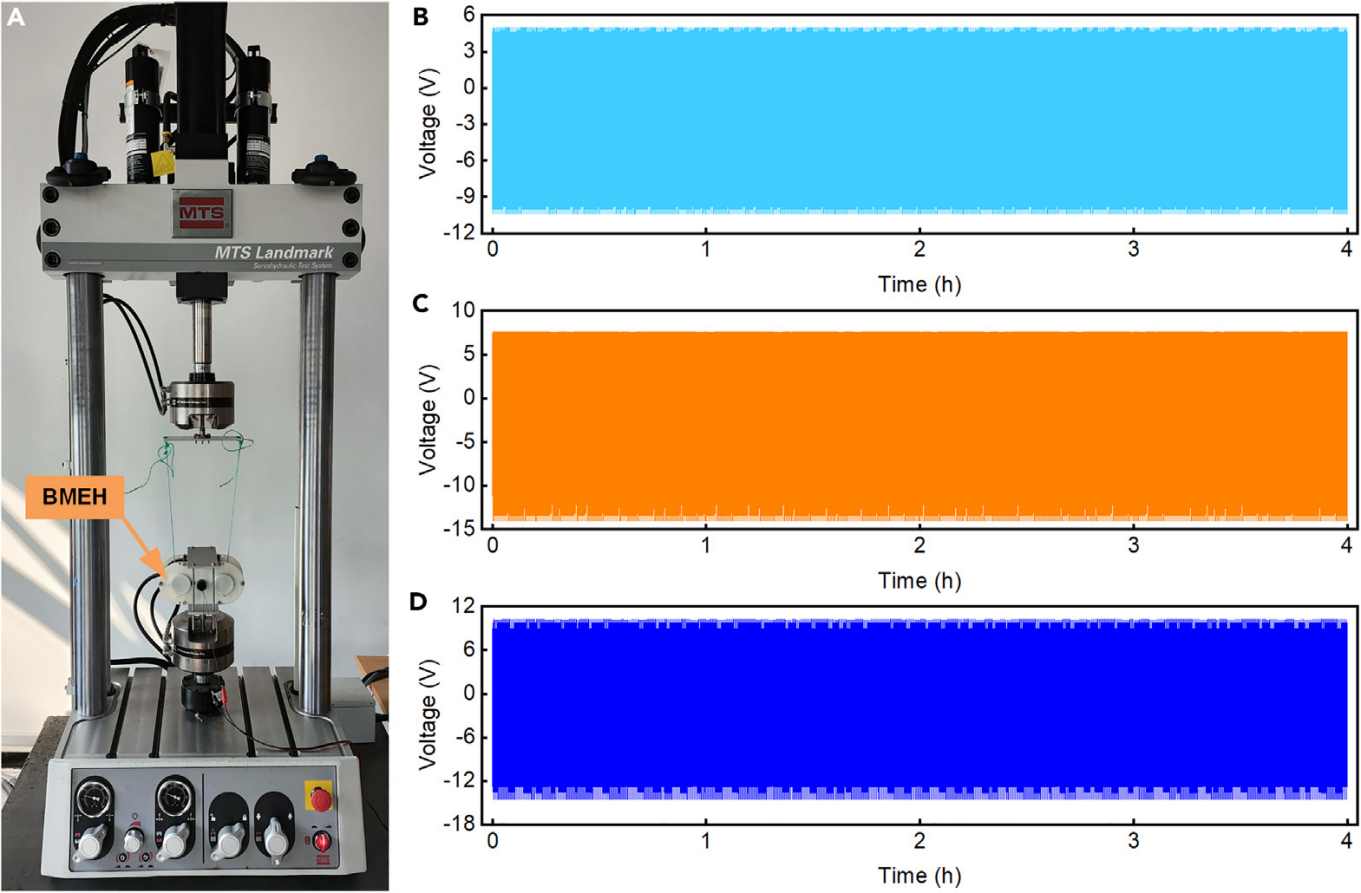
Fatigue experiments of BMEH (A) Fatigue experiments setup. (B) Voltage output of BMEH under excitation at a frequency of 0.5 Hz and an amplitude of 10 mm. (C) Voltage output of BMEH under excitation at a frequency of 1 Hz and an amplitude of 20 mm. (D) Voltage output of BMEH under excitation at a frequency of 1.5 Hz and an amplitude of 30 mm.

To verify the stability of the BMEH, fatigue experiments were conducted. Due to limited physical strength and other reasons, a person cannot maintain consistent motion for a long period of time, therefore mechanical testing & simulation (MTS) was utilized as the motion excitation input to test the reliability of the BMEH for continuous and long-time operation, as shown in Figure 9A. In the experiments, the excitation of the MTS was set to three different combinations. These were a frequency of 0.5 Hz and an amplitude of 10 mm, a frequency of 1 Hz and an amplitude of 20 mm, and a frequency of 1.5 Hz and an amplitude of 30 mm. The BMEH was operated for 4 h under each of the three excitations. (Figures 9B‒9D) show the voltages of the BMEH under different excitations. It can be found that the peak voltages of the BMEH are basically stable under three different excitations, which are 5 V, 7.6 V, and 10.2 V. The fact that the BMEH still maintains a stable voltage output under long-term excitation proves that it has good reliability and stability.

### Excitation monitoring of BMEH based on deep learning

**Figure 10:**
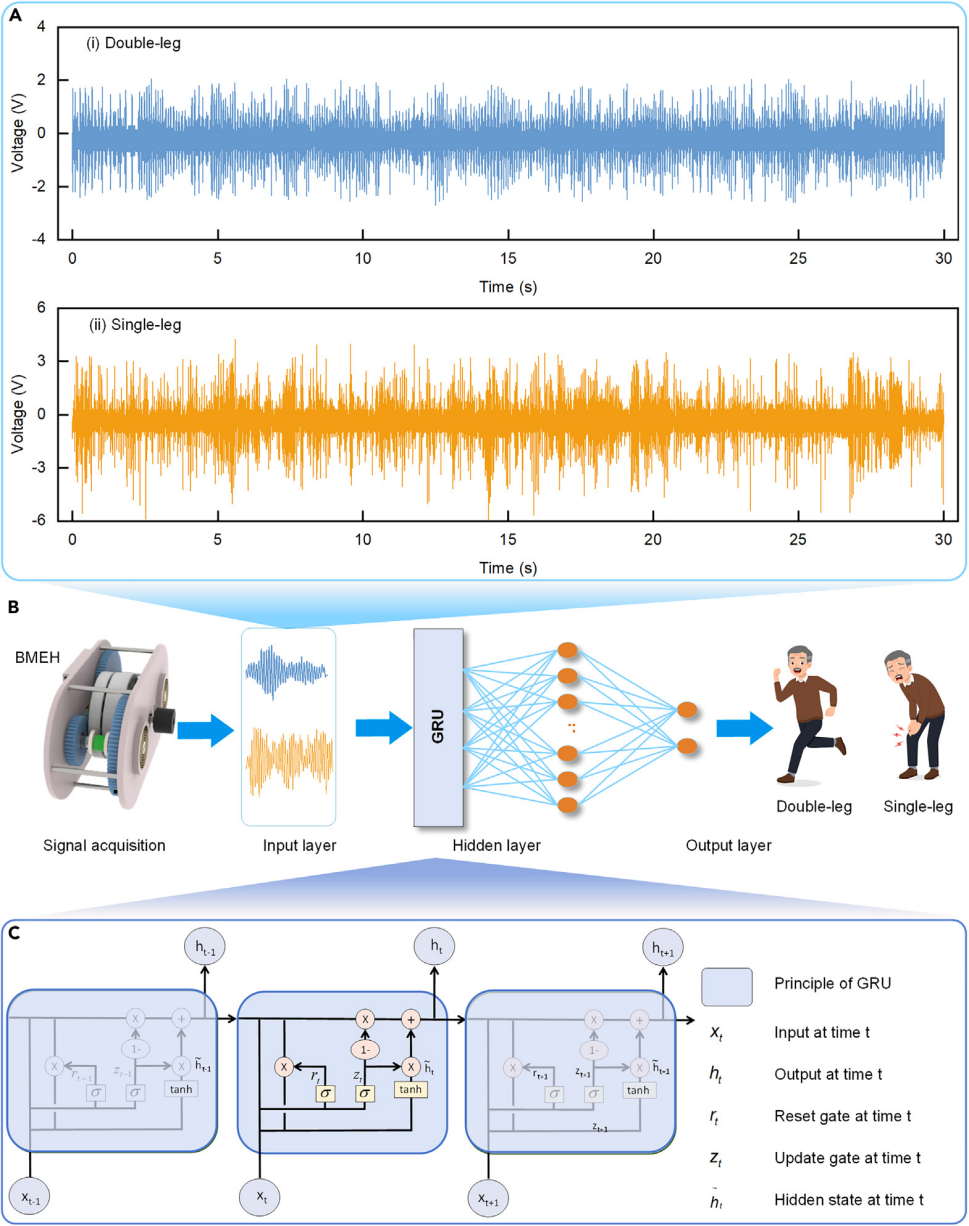
GRU deep learning model for excitation monitoring of BMEH (A) Voltage waveforms of the BMEH under different excitations of the double-leg and the single-leg. (B) The processing flow of motion recognition. (C) The principle of the GRU model.

The designed BMEH recovers negative energy from the knee joints of both legs as well as one leg. Usually, people walk with both legs, but in some special scenarios, they mainly rely on one leg to walk. The BMEH proposed in this study can not only recover the knee negative energy generation, but also use deep learning to realize the recognition of the excitation mode by analyzing the difference of the waveforms of the BMEH under different excitations of the single-leg and the double-leg. As demonstrated in Figure 10A, there are some obvious differences between the voltage waveforms generated by BMEH with single-leg excitation and double-leg excitation. There are many possible scenarios in which a person mainly relies on one leg to walk. The first may occur in medical rehabilitation. Recognizing walking styles can help rehabilitation professionals assess a patient’s gait and track rehabilitation progress. This is important for personalizing rehabilitation treatments and rehabilitation programs. Second is the field of smart health monitoring. In the home environment, recognizing walking styles can be used to monitor activity levels and walking habits of older adults or individuals with specific health problems. This can help provide alerts and support to ensure their safety. Then there is in sports and sports analytics. Recognizing walking models can be used to analyze an athlete’s gait and skills, providing feedback to coaches and athletes to improve training and performance. Finally, it is also relevant in security and surveillance. In the field of security and surveillance, recognizing walking models can be used to identify unusual activities or behaviors, helping security teams monitor and respond to potential risks.

We built a Gate Recurrent Unit (GRU) deep learning model, as shown in Figure 10. We collected 10 min of data for each BMEH under different excitations of the single-leg and the double-leg at a frequency of 2000. After preliminary sieving, we selected and intercepted the usable signals among them. The signals were simply preprocessed and cut with 2000 as the window length and 10 as the step, and ultimately obtained a total of 50,572 single-leg samples and 43,137 double-leg samples. The samples are then disrupted to produce a training set and a test set in a ratio of 8:2, where 10% of the test set is divided into a calibration set during network training. Figure 10B shows the network structure and data flow. The human body wears the BMEH for generating the electrical signal data, and the data are simply preprocessed and fed into the GRU-based network to discriminate the state of the subject. Through a large number of tuning experiments, the number of GRUs, the number of neurons in the fully connected layer, and the number of output neurons were selected to be 10, 16, and 2, respectively. Figure 10C shows the principle of the GRU. The internal reset gate and update gate can effectively retain and enhance the valid information in the array, and this module is often used for temporal array processing.

**Figure 11:**
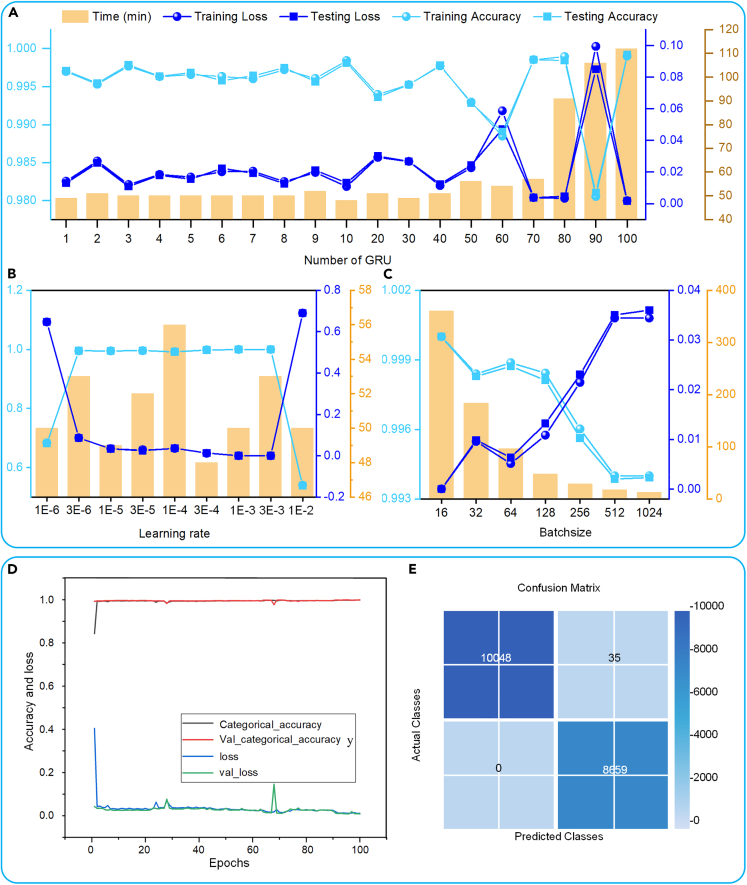
Experimental procedure and results of excitation monitoring based on GRU deep learning model (A) Experiment on the number of GRUs. (B) Effect of learning rate on the recognition rate of the network with a fixed number of GRUs and batchsize. (C) Effect of batchsize on the network at a fixed number of GRUs and learning rate. (D) Training process of the network. (E) Confusion matrix for the motion recognition.

To achieve a better network performance, we conducted some tuning experiments, and the experimental procedure and results are shown in Figure 11. The experimental software environment utilizes Python version 3.8 and TensorFlow version 2.6.0. The hardware setup consists of an Intel(R) Xeon(R) Silver 4210R CPU @ 2.40GHz 2.39 GHz processor and an NVIDIA GeForce RTX 3090 graphics card. First, the network is trained by varying the number of GRUs with fixed learning rate and batchsize, and the experimental results are shown in Figure 11A. It can be found that the training time and accuracy of the network vary less between the numbers of GRUs from 1 to 10, and the shortest time and highest accuracy are achieved at 10 GRUs. When the number of GRUs exceeds 10, the network training time gradually increases. Therefore, the number of GRUs is 10 is a more reasonable parameter. Figure 11B shows the analysis of the effect of the learning rate on the recognition rate of the network with a fixed number of GRUs and batchsize. The results show that the recognition rate of the network is close to 1 in the middle 7 sets of experiments, but the time taken is shortest at 0.0003, thus a learning rate of 0.0003 is desirable. Figure 11C shows the effect of batchsize on the network at a fixed number of GRUs and learning rate. It can be found that as the value of batchsize gradually becomes larger, the training time of the network is gradually shortened, but the accuracy of the network gradually decreases. After comparative analysis, it is determined that 128 is a more reasonable parameter for batchsize. Figure 11D illustrates the training process of the network. The number of GRUs is 10, the learning rate is 0.0003, and the batchsize is 128. It can be found that the accuracy curve and the loss curve of the calibration set and the two curves of the training set are basically overlapped, which proves that the network effectively extracts the features of the dataset. Figure 11E presents the performance of the network on the test set, and the results show that the network can achieve 99.8% accuracy. This proves that the proposed network can accurately achieve the recognition of the excitation mode through the electrical signals generated by the BMEH.

### Power generation assessment and application prospect of BMEH

Human activities are irregular, especially those who work outdoors for long hours. To evaluate the power generation of wearing a single BMEH in a single day, we assumed that outdoor workers walk or jog for 7 h a day, 1 h each at 1 km/h, 2 km/h, 3 km/h, 4 km/h, 5 km/h, 6 km/h, and 7 km/h, respectively. The average of the test results of the four testers was used as the power generated at different speeds. It was calculated that a single person wearing a single BMEH could generate 1060.85 J of energy in a single day.

Some other wearable devices such as heart rate monitoring sensor have low power, and most power consumption is only at the micro-watt level. Although no practical experiments have been conducted, the power generated by BMEH is enough to power other wearable devices. Table 3 shows more sensor types and their power. The time for which the daily power generation of one BMEH can meet the power consumption time of a single application is shown in fourth column. It can be seen from Table 3 that the proposed BMEH can be used as a power supply for low-energy-consumption wearable devices.

**Table 3:** Typical sensor types and their power

Type	Power (mW)	Company	Power supply time (h)
Heart rate monitoring sensor	4	TELESKY	73.67
Blood pressure sensors	5	SICHIRAY	58.94
Temperature sensor	1.8	ZAVE	163.71
Breathing sensors	3.2	SICHIRA	92.09
Humidity sensor	0.2	RISYM	1473.4

**Figure 12:**
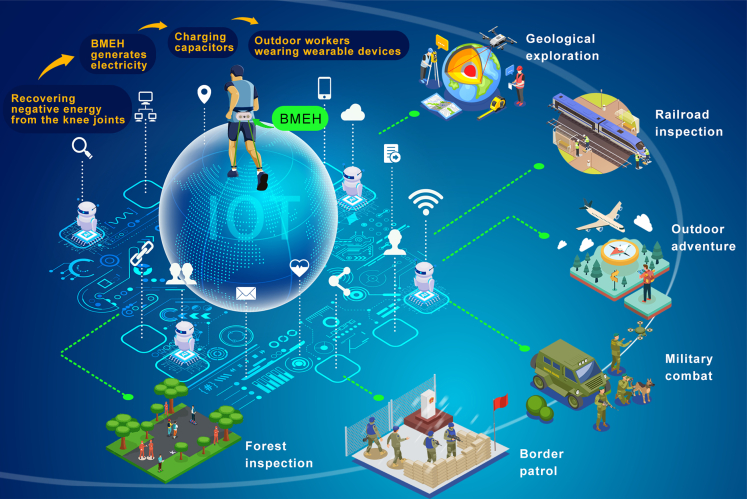
The application diagram of the designed BMEH

Wearable devices have a variety of functions, including recording exercise data, detecting health data, reminder function, navigation function, social function, and so on. With the development of AI and IoT, wearable devices will be applied to more and more scenarios. Especially for people who work outdoors for a long time, wearable devices are important helpers for their work. As shown in Figure 12, BMEH-powered wearable devices are expected to be used in geological exploration, military combat, border patrols, outdoor adventures, and more.

### Conclusions

To prolong the endurance of wearable devices, we designed a BMEH to recover negative energy from the knee joints to generate electricity. The proposed BMEH achieves the self-sustainable of powering wearable devices and sensing. The HARE principle of HEH design including human-friendliness, adaptability, reliability, and economy is first proposed. A BMEH is then designed based on the HARE principle, which recovers only the negative energy of knee joints for power generation.

The proposed BMEH includes a motion conversion module and an electricity conversion module. The motion conversion module converts the ultra-low frequency knee extension motion into the high-frequency rotation motion of the double-rotor. The electricity conversion module based on Halbach magnet arrays generates electricity. The voltage regulator and rectifier circuits convert the unstable AC power generated by the BMEH to stable DC power for wearable devices. The BMEH is mounted on the waist of the human body and connected to the calves through ropes. The extension motion of the knee joint is transformed into a rotational motion. The rotation motion is accelerated by the acceleration gear set, and finally the half-wave rectification mechanism is used to realize the high-speed rotation of the double-rotor.

We selected four testers with different heights and weights to wear the BMEH and investigated the output performance of the BMEH. At a speed of 7 km/h, the average output power of BMEH is 66.85 mW, and the output power density reaches 0.07 W/kg. The experimental results show that the power generated by the tester wearing BMEH walking at 4 km/h can light up the LED strip, charge the bracelet, and charge the temperature and humidity sensor. Based on the evaluation of the experimental results, the daily power generation of the single BMEH can reach 1060.85 J.

In addition, we build a GRU deep learning model for analyzing and extracting the output electrical signal characteristics of BMEH under single-leg and double-leg excitation. The optimal parameters of the GRU model were determined by parameter optimization analysis. Ultimate results show that the BMEH demonstrates the excitation mode detection accuracy of 99.8% based on the GRU GRU deep learning model.

### Limitations of the study

Although we have confirmed the feasibility of the proposed BMEH by calculations and experiments, etc., there are still some aspects that can be improved or are worth studying at a later stage: (i) the magnets used in this design are N35, and by replacing it with better magnets, such as N52, the power generation of BMEH will be substantially increased; (ii) determining and increasing power density. Optimization of coil parameters, including coil thickness, wire diameter, etc., to further enhance the power density of BMEH; (ii) the COH value of BMEH needs to be further investigated after the experimental conditions have been improved; (iii) further optimization of the ratio of the accelerating gear set to enhance energy utilization efficiency; and (iv) the human activity is complex and variable, and the real power generation performance of the proposed BMEH needs to be tested on outdoor workers.

## STAR★Methods

### Key resources table

**Table:** 

REAGENT or RESOURCE	SOURCE	IDENTIFIER
**Software and algorithms**		

Microsoft Visio 2019	Microsoft	https://www.microsoft.com/zh-cn/microsoft-365/visio/flowchart-software
Origin 2021	Originlab	https://www.originlab.com/
COMSOL Multiphysics 6.1	COMSOL	https://cn.comsol.com/
Python 3.8	Python	https://www.python.org/
TensorFlow 2.6.0	TensorFlow	https://tensorflow.google.cn/?hl=zh-cn

**Other**		

DS1102Z-E digital oscilloscope	RIGOL	https://rigol.com
Resistor box	SHANE ZX99-IA	http://www.sy-siac.cn/index.html

### Resource availability

#### Lead contact

Further information and requests for resources and reagents should be directed to and will be fulfilled by the lead contact Zutao Zhang (zzt@swjtu.edu.cn).

#### Material availability

This study did not generate new unique reagents.

#### Data and code availability

• All data reported in this paper will be shared by the lead contact upon reasonable request.
• This paper does not report original code.
• Any additional information required to reanalyze the data reported in this paper is available from the lead contact upon request.

### Experimental model and study participant details

The testers are adult male in good health with normal level of movement. There are no physical or physiological discomfort after completion of the experiment. The testers provided written informed consent prior to participation. The experimental procedures were approved by the School of Mechanical Engineering, Southwest Jiaotong University. The experiment took 120 to 150 minutes to complete and participants were compensated for their time (200 RMB).

### Method details

All methods can be found in the manuscript. Please check the **Structure and working principle of BMEH** for structural design. Please check the **MODELLING AND ANALYSIS** for theoretical models and simulations of the system. Please check the **EXPERIMENTS SETUP** for prototyping and setting up experiments. Please check the **Power generation assessment and application prospect of BMEH** to know the application potential of the device. Using Microsoft Visio 2019 to generate visual images in the manuscript. Origin 2021 was used to process experimental data and generate visual images in the manuscript. The dynamic modeling software COMSOL Multiphysics 6.1 was used to simulate the magnetic field distribution. Python 3.8 and TensorFlow 2.6.0 are used to utilize the experimental software environment. The oscilloscope (RIGOL DS1102) is used to evaluate the output characteristics of the equipment. A resistor box (SHANE ZX99-IA) is used to provide external load. Python version 3.8 and TensorFlow version 2.6.0 are used to utilize the experimental software environment of deep learning. In addition, a presentation video is provided to introduce the whole paper, as shown in Video S1.

Video S1. Related to STAR Methods

### Quantification and statistical analysis

Microsoft Visio 2019 is used to generate the visual images in the manuscript. Origin 2021 is used to process experimental data and generate visual images in the manuscript. The dynamic modeling software COMSOL Multiphysics 6.1 is used to simulate magnetic flux distribution and magnet force. The voltage signals are captured by the digital oscilloscope (RIGOL DS1102). Python version 3.8 and TensorFlow version 2.6.0 are used to utilize the experimental software environment of deep learning.
